# Bacterial interference with canonical NFκB signalling

**DOI:** 10.1099/mic.0.069369-0

**Published:** 2013-10

**Authors:** Mona Johannessen, Fatemeh Askarian, Maria Sangvik, Johanna E. Sollid

**Affiliations:** Research Group of Host–Microbe Interactions, Department of Medical Biology, Faculty of Health Sciences, University of Tromsø, Tromsø, Norway

## Abstract

The human body is constantly challenged by a variety of commensal and pathogenic micro-organisms that trigger the immune system. Central in the first line of defence is the pattern-recognition receptor (PRR)-induced stimulation of the NFκB pathway, leading to NFκB activation. The subsequent production of pro-inflammatory cytokines and/or antimicrobial peptides results in recruitment of professional phagocytes and bacterial clearance. To overcome this, bacteria have developed mechanisms for targeted interference in every single step in the PRR–NFκB pathway to dampen host inflammatory responses. This review aims to briefly overview the PRR–NFκB pathway in relation to the immune response and give examples of the diverse bacterial evasion mechanisms including changes in the bacterial surface, decoy production and injection of effector molecules. Targeted regulation of inflammatory responses is needed and bacterial molecules developed for immune evasion could provide future anti-inflammatory agents.

## Introduction

Micro-organisms colonize all available surfaces of the human body. The immune sentinels of the skin and mucosa recognize both commensals and pathogens with the intention to dampen colonization and potential invasion. Highly conserved bacterial structures, termed pathogen associated molecular patterns (PAMPs) are recognized by sensors such as pattern-recognition receptors (PRRs). Among the PRRs are the Toll-like receptors (TLRs), nucleotide oligomerization domain (NOD)-like receptors (NLRs), RIG-I-like receptors (RLRs) and C-type lectin receptors (CLRs). A microbe can be recognized by independent PRRs simultaneously, and the sensors activate various signal cascades that generally converge to transcription factors including NFκB, activator protein (AP)-1, nuclear factor of activated T cells (NFAT) and interferon regulatory factors (IRFs), which then activates transcription of immunity-related genes (Nish & Medzhitov, 2011; Lee & Kim, 2007).

Bacteria have evolved various immune evasive mechanisms such as prevention of complement deposition, blockade of antibody function and dampening of neutrophil recruitment by production of decoy molecules that disturb the chemotaxic gradient (Bestebroer *et al.*, 2010; Veldkamp & van Strijp, 2009). Central in the first line of defence against bacteria is the recognition of the bacteria by the PAMP–PRR. However, different bacteria, both pathogenic and non-pathogenic, vary in the ability to induce production of immunity-related molecules (Wanke *et al.*, 2011; Neish *et al.*, 2000). This may be explained by species- or strain-dependent variation in ability to interfere with early host cell signalling. Such an early inhibitory mechanism may be of importance for survival as a commensal or in promoting bacterial infection due to decline in the first induced pro-inflammatory responses (Ashida *et al.*, 2010). In this review, we aim to give some examples of how bacteria can modulate their recognition by interfering with canonical host cell signalling to NFκB.

### Canonical NFκB signalling and the immune response

NFκB proteins are a family of five structurally related transcription factors: RelA/p65, RelB, c-Rel and the proteins synthesized as precursors, p50 (p105/NFκB1) and p52 (p100/NFκB2). They all contain a conserved Rel homology domain (RHD) that contains a nuclear localization signal (NLS), directing the transcription factors into the nucleus. Moreover, the RHD is involved in the homo- or heterodimerization of the transcription factors, and in their binding to DNA at κB sites that are localized within target gene promoters (Li & Verma, 2002).

**Fig. 1.**
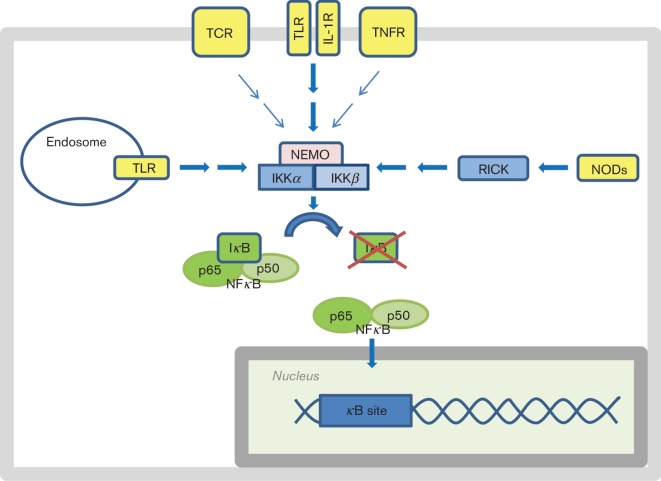
Activation of the canonical signalling pathway to NFκB results in nuclear translocation of the transcription factor. NFκB is sequestered in the cytosol in resting cells due to binding to IκB. IκB can be degraded (illustrated by the red cross) upon signalling from the activated NEMO/IKKα/IKKβ complex (IKK complex). The IKK complex can be activated by various stimuli that engage the TCR, TLRs, IL-1R, TNFR and NLRs.

NFκB is sequestered in the cytosol by inhibitor of κB (IκB) in resting cells, and appropriate stimuli are needed to induce its translocation to the nucleus. The canonical signalling pathway to NFκB is induced by stimuli that activate the tumour necrosis factor receptor (TNFR), the interleukin-1 receptor (IL-1R), the T-cell receptor (TCR), TLRs and the NLRs NOD1 and NOD2 (Oeckinghaus *et al.*, 2011; Sun, 2012). All the aforementioned receptors use various adaptors and signalling molecules, but the signalling converges at the IκB kinase (IKK) complex containing IKKα, IKKβ and NFκB essential modifier (NEMO), upstream of IκB and NFκB (Oeckinghaus *et al.*, 2011; Sun, 2012) (Fig. 1).

**Fig. 2.**
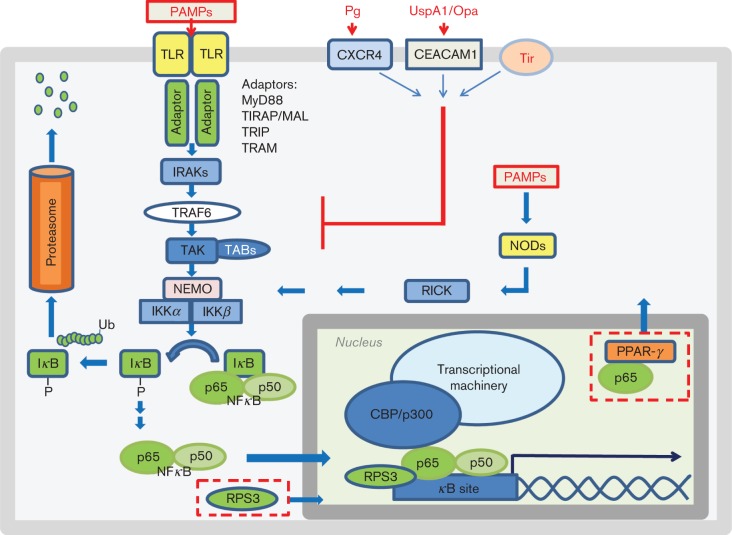
Engagement of TLRs or NODs by PAMPs results in NFκB activation. PAMPs induce conformational changes in TLRs, resulting in recruitment of appropriate adaptors for example MyD88/MAL. The adaptors recruit the IRAKs which autophosphorylate thereby attracting TRAF6 which again activates the TAK complex resulting in stimulation of the NEMO/IKKα/IKKβ complex (IKK complex). The activated IKK complex phosphorylates IκB, targeting it for ubiquitination and proteasomal degradation. NFκB can then enter the nucleus where it binds to the κB site on appropriate promoters, and the binding can be improved by RPS3. The TAD of p65 recruits co-activators such as CBP/p300 which results in establishment of the transcriptional machinery and transcription of genes encoding proteins involved in immunity. The bacterial surface structure proteins Pg-fimbriae and UspA1/Opa stimulate CXCR4 and CEACAM1 receptors thereby activating cascades that negatively interfere with TLR-induced NFκB activation. Injected *E. coli* Tir protein inhibits the cascade with a similar mechanism. Nuclear proteins can also be targeted by bacterial effectors. Bacterial effectors can prevent translocation of RPS3 into the nucleus. Similarly, bacterial presence can induce PPAR-γ-mediated nuclear translocation of p65. In the figure, bacterially derived molecules are written in red. The bacterially induced negative interference in signalling pathways or localization of cellular proteins is illustrated with red lines or boxes with red dashed lines, respectively. Ub, ubiquitin; P, phosphate; CBP/p300, CREB binding protein/p300; TAD, transactivation domain.

Ten members of the TLR protein family are found in humans and include the cell membrane-bound TLR1, TLR2, TLR4, TLR6 and the endosome-associated TLR3, TLR7, TLR8 and TLR9 (Casanova *et al.*, 2011; Nestle *et al.*, 2009). TLRs belong to the Toll-like/IL-1 receptor superfamily that shares an intracellular domain referred to as the Toll/interleukin-1 receptor (TIR) domain. Engagement of TLRs by PAMPs activates an intracellular cascade resulting in induction of pro-inflammatory genes (Fig. 2). More specifically, the PAMP–TLR interaction results in receptor-dimerization via the TIR domain, and/or a conformational change. The TIR domain of the receptors then attracts TIR- containing adaptor proteins such as myeloid differentiation factor 88 (MyD88), MyD88-adaptor like (MAL, also called TIRAP), TIR-domain-containing adaptor protein including interferon-β (TRIF), TRIF-related adaptor molecule (TRAM) or sterile α- and armadillo-motif-containing protein (SARM). TLR recruitment of the adaptors, except SARM, activates the signal cascade that subsequently recruits downstream signalling molecules (Watters *et al.*, 2007). MyD88 either binds TLRs directly (TLR5, TLR7, TLR8, TLR9) or indirectly (TLR1, TLR2, TLR4, TLR6) through MAL/TIRAP (O’Neill & Bowie, 2007). MyD88 comprises an N-terminal death domain (DD) and a C-terminal TIR-domain, separated by a small intermediate domain (ID) (Watters *et al.*, 2007). MyD88 interacts with TLRs via the TIR domain, while the DD of MyD88 associates with the DD of the IL-1R-associated kinases IRAK1, IRAK2 and IRAK4. The kinases are then activated due to auto- and cross-phosphorylation. This leads to recruitment of tumour necrosis factor receptor-associated factor (TRAF)6, followed by a complex consisting of growth factor-β-associated kinase- 1 (TAK1), TAK1 binding protein (TAB)1 and TAB2. TAK1 can phosphorylate IKKβ, leading to activation of the IKK complex. Next, the IKK complex phosphorylates IκB, which then is recognized by a subunit of the ubiquitin E3 ligase complex SCF^βTrCP^. This leads to K48-polyubiquitination of IκBα, which targets it for degradation by the proteasome (Jiang & Chen, 2012; Watters *et al.*, 2007; Chen, 2005). When IκBα is degraded, the NLS of NFκB subsequently becomes available, and the transcription factor can translocate to the nucleus. In the nucleus NFκB can bind to κB sites, thereby influencing gene expression with impact on immune responses (Sun, 2012; Newton & Dixit, 2012; Kumar *et al.*, 2009) (Fig. 2).

Activation of TLRs and other PRRs are associated with the risk of chronic inflammatory diseases and immune disorders, and the pathway needs to be highly regulated. Endogenous inhibition of TLR signalling can occur at various levels. The level of TLRs can be reduced either by increased degradation or by reduced production, or the receptor-induced signalling can be inhibited by e.g. the host encoded orphan receptor ST2 (Jeong & Lee, 2011; Han & Ulevitch, 2005). Inhibition of the pathway can also be induced. For example, during chronic conditions of inflammation or prolonged exposure to LPS, a splice variant of MyD88 named MyD88s is produced. MyD88s acts as a negative regulator of MyD88 function and blocks its signalling (Burns *et al.*, 2003; Janssens *et al.*, 2002). Similarly, IRAK-M is another example of an endogenously produced antagonist of TLR pathways that inhibits signalling at the level of IRAKs (Han & Ulevitch, 2005).

The importance of TLR signalling in immune defence can also be observed by studying naturally occurring genetic mutations or polymorphisms in innate immune genes among humans. Since 2003, 24 patients with MyD88 deficiencies and 52 patients with IRAK4 deficiencies have been followed. The deficiencies are life-threatening during childhood, and the paediatric patients have a predisposition to severe bacterial infection often caused by pyogenic Gram-positive bacteria, such as *Streptococcus pneumoniae* and *Staphylococcus aureus* (von Bernuth *et al.*, 2012). Similar effects are seen in patients with mutations that abrogate the genes encoding NEMO and IκBα (Suhir & Etzioni, 2010; Turvey & Hawn, 2006). Also, certain polymorphisms in TLRs or signalling components affect susceptibility to infection. Humans with TLR2 Arg753Gln show increased susceptibility to tuberculosis and infections by Gram-positive organisms. Similarly, individuals with TLR4 Asp299Gly or Thr399Ile are hypo-responsive to LPS (Carpenter & O’Neill, 2007).

These studies demonstrate that PRR- and MyD88-induced signalling is of importance in protection against various bacteria especially during childhood. Therefore, the presence of immune evasion mechanisms targeting this signalling pathway is not surprising. This strategy may allow the bacteria to survive as commensals (Xavier & Podolsky, 2000), or to stay un-recognized for as long as possible during infection.

### Bacterial evasion mechanisms targeting PRR–NFκB signalling

#### Evasion of recognition by pattern recognition receptors.

One can imagine several mechanisms which could interfere with TLR signalling. One approach is to avoid the recognition of PAMPs, exemplified by a mechanism involving a polysaccharide capsule on the outermost surface of the bacteria. In the case of *Enterococcus faecalis*, the complement factor C3 is deposited on the surface of capsular serotypes C and D, as well as on the non-encapsulated strain. However, the presence of the capsule masked the complement from detection by anti-C3 antibodies and protected against opsono-phagocytosis. Similarly, the PAMPs LTA and peptidoglycan were masked from detection by antibodies in encapsulated strains, and these strains showed reduced TNF-α production by the macrophage cell line RAW264.7 (Thurlow *et al.*, 2009). A similar mechanism is used by *Staph. aureus* (Nanra *et al.*, 2012; O’Riordan & Lee, 2004). The efficiency of masking the bacterial cell surface may be dependent on the particular capsule type expressed, as for example the capsule of *Strep. pneumoniae* type 3 strain WU2 masked surface antigens more effectively than the type 2 D39 strain (Abeyta *et al.*, 2003).

A second approach is to modify the surface of PAMPs, thereby interfering with their recognition by pattern recognition receptors such as TLRs or NODs. This strategy is used, for example, by *Salmonella typhimurium*. Presence of the host is recognized by the bacterial sensor histidine kinase PhoP localized on the bacterial surface, which then phosphorylates the intracellular effector molecule PhoQ which results in bacterial expression of lipid A modifying enzymes. The modified version of lipid A has reduced ability to induce TLR4-mediated NFκB-activation (Kawasaki *et al.*, 2004). *Pseudomonas aeruginosa* also modifies its lipid A and muropeptide during chronic cystic fibrosis infection, resulting in reduced cytokine secretion and leukocyte recruitment (Cigana *et al.*, 2009). Similar strategies are used by *Francisella tularensis* and *Mycobacterium tuberculosis*. The lipid A of the former is hypo-acetylated (tetra-acetylated) and not properly recognized by TLR4 (Gunn & Ernst, 2007), while the cell envelope-associated serine hydrolase Hip1 of *My. tuberculosis* restricts the onset and magnitude of pro-inflammatory cytokines probably by limiting recognition of TLR2 ligands (Madan-Lala *et al.*, 2011). When the bacteria are inside the host cell, peptidoglycan breakdown products become available for detection by the PRRs NOD1 and NOD2. NOD engagement of PAMPs activates receptor interacting serine/threonine kinase (RICK), resulting in activation of NFκB-mediated transcription (Kersse *et al.*, 2011) (Fig. 2). Several bacteria modify peptidoglycan in order to avoid being recognized by NODs (Wolfert *et al.*, 2007), and *Legionella pneumophila* encodes a periplasmic protein EnhC that inhibits degradation of peptidoglycan thereby preventing production of PAMPs (Liu *et al.*, 2012).

A third approach is to inhibit TLR signalling by direct interactions between secreted bacterial effector molecules and the host TLR receptors. *Staph. aureus* may express and secrete staphylococcal superantigen-like proteins (SSLs) and several of the SSLs have immune modulating properties (Fraser & Proft, 2008). Recently SSL3, and to a certain extent SSL4, were found to negatively interfere with TLR2-mediated production of IL-8 (Bardoel *et al.*, 2012). The inhibitory effect is most likely due to competition between SSL3 and the PAMP ligands of TLR2. The inhibition is partly dependent on sialic residues on TLR2 (Bardoel *et al.*, 2012; Yokoyama *et al.*, 2012).

A fourth approach is to activate a counteracting signalling pathway which negatively interferes with the TLR-signalling cascade (Fig. 2). *Porphyromonas gingivalis* has surface bound fimbriae often referred to as Pg-fimbriae. Pg-fimbriae can interact with TLR2/CD14 resulting in increased NFκB activity. However, Pg-fimbriae simultaneously interact with an additional host receptor, CXC-chemokine receptor 4 (CXCR4), resulting in intracellular cross-talk between two different signalling pathways (e.g. the cAMP/PKA pathway and the TLR2 pathway) which leads to impaired NFκB activation and host defence (Hajishengallis *et al.*, 2008). *Moraxella catarrhalis* and *Neisseria meningitidis* use a similar strategy with their ubiquitous surface protein A1 (UspA1) and opacity-associated protein (Opa), respectively. These structurally unrelated proteins can both interact with the Ig-like CEACAM1 receptor of host cells (Slevogt *et al.*, 2008). The intracellular part of CEACAM1 composes an immune-receptor tyrosine-based inhibition motif (ITIM). When ITIM-bearing receptors engage their ligands, intracellular tyrosine residues becomes phosphorylated resulting in recruitment of phosphotyrosine phosphatases such as SHP-1, SHP-2 or the inositol phosphatase SHIP. The TLR adaptors TIRAP and MyD88 can be tyrosine phosphorylated, suggesting that ITIM can have a regulatory role in TLR signalling (Barrow & Trowsdale, 2006). Indeed, the interaction between bacterial UspA1/Opa and host CEACAM1 results in recruitment of an intracellular phosphatase which negatively regulates TLR2-dependent activation of the NFκB pathway (Slevogt *et al.*, 2008). Similarly, *Staph. aureus* is found to interact with paired Ig-like receptors containing ITIMs on mouse macrophages and thereby negatively interfere with TLR-mediated cytokine production in these cells (Nakayama *et al.*, 2007). In either case, the net result of the interaction with ITIM-containing receptors is that activation of NFκB is inhibited. The above-mentioned examples include bacterial cell surface proteins that interact with an ITIM-containing host receptor. In contrast, enteropathogenic *Eschericia coli* (EPEC) uses a strategy where it injects bacterial intimin receptor (Tir) into the epithelial cell membrane. The extracellular part of Tir is engaged by the bacterial surface ligand intimin, while the intracellular part of Tir contains a region with similarity to host ITIMs (Yan *et al.*, 2012). The ITIMs of the bacterial Tir protein interact with the eukaryotic tyrosine phosphatase SHP-1, and enhance its binding to TRAF6. The ubiquitination and activation of TRAF6 is inhibited, and EPEC-induced expression of pro-inflammatory cytokines is suppressed (Yan *et al.*, 2012) (Fig. 2).

#### How to prevent recruitment and activity of the adaptor molecules?

Activated Toll-like receptors recruit the appropriate intracellular adaptors MyD88, MAL/TIRAP, TRIF or TRAM (Newton & Dixit, 2012; Takeda & Akira, 2004) (Fig. 2). One mechanism to evade signal transduction at this stage is to interfere with the stability of the relevant adaptor proteins. *Brucella* spp. encode a protein named TcpB/Btp1 that negatively interferes with NFκB-activity *in vitro* (Cirl *et al.*, 2008; Radhakrishnan *et al.*, 2009; Salcedo *et al.*, 2008; Sengupta *et al.*, 2010). TcpB mimics the membrane binding properties of TIRAP/Mal and localizes to the cellular plasma membrane (Radhakrishnan *et al.*, 2009). TcpB can also interact with the adaptor MAL/TIRAP, thereby inducing its ubiquitination and degradation (Sengupta *et al.*, 2010), and this prevents host signal transduction.

All the TLRs, IL-1R and the adaptors MyD88, TIRAP/Mal, TRIF, TRAM and SARM share an intracellular conserved region called the TIR domain (Watters *et al.*, 2007). The TIR domain is approximately 200 amino acids in length and sequence conservation is generally in the 20–30 % range (Xu *et al.*, 2000). Crystallographic analysis of the TIR domains of human TLR1, TLR2 and TLR10 has shown that the TIR-domain is composed of five β-sheets (βA–βE) surrounded by five α-helices (αA–αE). The loop connecting βB and αB, named the BB-loop, forms a protrusion that extends from the surface of the TIR domain. Three conserved amino acid motifs named boxes 1, 2 and 3 exist, and the BB-loop comprises a part of box 2, which is of importance in eukaryotic signal transduction (Li *et al.*, 2005; Xu *et al.*, 2000; Watters *et al.*, 2007). Indeed, replacement of proline 712 in TLR4 or proline 681 in TLR2 with histidine results in receptors that do not respond to LPS or Gram-positive bacteria (Hoshino *et al.*, 1999; Poltorak *et al.*, 1998; Underhill *et al.*, 1999). In the case of TLR2, studies have shown that the mutation P681H prevents its interaction with MyD88, thereby blunting further signal transduction (Xu *et al.*, 2000). The TIR–TIR interaction is therefore of importance in signalling, and another immune evasion strategy is to interfere with this. Indeed, Cirl and coworkers found that the uropathogenic *E. coli* CTF073 encodes a TIR-containing protein named TcpC, which via its binding to MyD88 negatively interferes with TLR4–MyD88–NFκB signalling (Cirl *et al.*, 2008; Yadav *et al.*, 2010). Urinary tract infection (UTI) studies in mice revealed that *E. coli* with TcpC could multiply to higher numbers than TcpC-deficient strains, and molecular epidemiological studies among human UTI patients revealed that TcpC was associated with severity of disease (Cirl *et al.*, 2008). An intact BB loop of the bacterial TIR domain is of importance for its function, e.g. the proline residue in the exposed BB loop of *Yersinia pestis* YpTIR was needed for the interaction with MyD88, as the Pro173His mutant of YpTIR did not interfere with LPS-induced signalling (Spear *et al.*, 2012). Similarly, mutation of a particular glycine in the BB-loop of *Brucella* TcpB resulted in reduced ability to suppress NFκB (Radhakrishnan *et al.*, 2009). Furthermore, database searches have shown that TIR domains are found in hundreds of bacterial species (Spear *et al.*, 2009; Zhang *et al.*, 2011a), and phylogenetic analysis reveals that SARM, which is the only TIR-containing adaptor that negatively regulates TLR signalling, is clustered together with bacterial TIR proteins (Zhang *et al.*, 2011a).

#### Can bacteria interfere with IRAKs, TRAFs or TAK/TAB complexes?

The TLR/MyD88 interaction makes the DD of MyD88 available for interaction with the DD of serine/threonine kinase IRAK4, which via its DD domain binds to the DD domain of the related kinases IRAK1 and IRAK2. MyD88 and IRAKs nucleate a larger complex, which in the case of TLR4 signalling includes TRAF6 (Newton & Dixit, 2012) (Fig. 2).

IRAK-M is an endogenous inhibitor of IRAK activity (Han & Ulevitch, 2005) and IRAK-M-deficient mice had reduced lethality after infection with *Strep. pneumoniae* (van der Windt *et al.*, 2012). Reduced lethality in the absence of the negative regulator IRAK-M reveals that inhibition of the pathway is beneficial for the microbe. One approach for a microbe to hijack signalling could be to induce expression of endogenous inhibitors in host cells. The mammalian intestinal tract harbours a large number of commensal bacteria which could also be recognized via their PAMPs. However, commensals were found to induce expression of IRAK-M in the intestine of BALB/c mice, and the authors suggest that the induction of a negative regulator of TLR signalling plays a role in homeostasis of the intestine and survival of the microbes (Biswas *et al.*, 2011). Induced IRAK-M expression is also found in THP-1-derived macrophages after exposure to LPS from *Po. gingivalis*. The resultant reduced production of the pro-inflammatory cytokines TNFα, IL-6 and IL-12p40 may be advantageous for the bacteria, causing chronic infection during periodontitis (Domon *et al.*, 2008).

The next level in the signalling pathway involves TRAF6 and TAB/TAK (Fig. 2). The function of the E3 ubiquitin ligase TRAF6 can also be a target for bacterial immune evasion. Ubiquitin is a conserved 76 amino acid peptide in eukaryotes that can be covalently attached to other proteins through an enzymatic process involving three sequential steps performed by one ubiquitin-activating enzyme (E1), ubiquitin-conjugating enzymes (E2s) and ubiquitin-ligating enzymes (E3s). Host proteins can be modified by addition of polyubiquitin chains, and K63-linked polyubiquitination is often involved in signal transduction. When induced, for instance due to the presence of a PAMP (Fig. 2), the signalling cascade results in activation of TRAF6 that performs K63-polyubiquitination of itself. This leads to recruitment of zinc-finger ubiquitin-binding proteins TAB2 and TAB3, which are regulators of the TAK1 complex (Jiang & Chen, 2012). One option for evasion of the cascade is to prevent polyubiquitination in the first place. The E3 ubiquitin ligase function of TRAF6 is dependent on E2 ubiquitin-conjugating enzymes such as UBC13. This particular E2 conjugating enzyme is a target for *Shigella flexneri* OspI, which deamidates the UBC13 glutamine residue at position 100 into glutamate. Consequently, the E2 conjugating activity which is required for TRAF6 activation is inhibited (Sanada *et al.*, 2012). Another option for evasion is to remove ubiquitin-chains that were already placed on target proteins. Indeed, *Yersinia pseudotuberculosis* and *Yersinina enterocolitica* encode a de-ubiquitinating protease named *Yersinia* outer protein J (YopJ). Bacterial YopJ in host cells results in removal of K63-polyubiquitination of TRAF6. As the K63-polyubiquitination of TRAF6 is a signal for recruitment of the NEMO/IKKα/IKKβ complex and the TAK1/TAB1/TAB2 complex, the YopJ induced de-ubiquitination blocks the host intracellular signalling to NFκB (Zhou *et al.*, 2005; Sweet *et al.*, 2007).

**Table 1.** Immune evasion mechanisms at various steps in the TLR–NFκB pathway

Targeted step in the pathway	Bacterial species	Bacterial molecule/effector	Mechanism of action	Reference
TLRs	*En. faecalis*	Capsule	Masks PAMPs from detection	Thurlow *et al.*, 2009
*Staph. aureus*	Capsule	Masks PAMPs from detection	Nanra *et al.*, 2012; O’Riordan & Lee, 2004
*Strep. pneumoniae*	Capsule	Masks PAMPs from detection	Abeyta *et al.*, 2003
*Ps. aeruginosa*	Lipid A	*pagL* mutation and modification of lipid A over prolonged infection	Cigana *et al.*, 2009
*Sal. typhimurium*	Lipid A	Induced expression of lipid A modifying enzymes	Kawasaki *et al.*, 2004
*My. tuberculosis*	Hip1	Limits the recognition of TLR2 ligands	Madan-Lala *et al.*, 2011
*Staph. aureus*	SSL3	Binds to and inhibits TLR2 function	Bardoel *et al.*, 2012; Yokoyama *et al.*, 2012
*Po. gingivalis*	Pg	Binds CXCR4, thereby counteracting TLR2 effect	Hajishengallis *et al.*, 2008
*Mo. catarrhalis*, *N. meningitides*	UspA1, Opa	Binds CEACAM1 and recruits SHP-1, which negatively interferes with signalling (dephosphorylates the p85α binding motif of TLR2)	Slevogt *et al.*, 2008
*E. coli*, *Citrobacter rodentium*	Tir	Bacterial receptor located in host membrane, recruits SHP-1 that negatively interferes with signalling (interferes with TRAF6 ubiquitination)	Yan *et al.*, 2012
TIRAP/MyD88	*Brucella melitensis*, *Br. abortus*	TcpB/Btp1	Mimics TIRAP, causing its degradation	Sengupta *et al.*, 2010; Radhakrishnan *et al.*, 2009
	*E. coli*	TcpC	Binds to MyD88 and negatively interferes with signalling	Cirl *et al.*, 2008
IRAKs	*Po. gingivalis*	LPS	Induces expression of the inhibitory IRAK-M	Domon *et al.*, 2008
TRAF	*Y. pestis*	YopJ	Prevents K63-polyubiquitination of TRAF6	Sweet *et al.*, 2007; Zhou *et al.*, 2005
	*Shig. flexneri*	OpsI	Deamidates and inactivates the ubiquitin-conjugating enzyme Ubc13	Sanada *et al.*, 2012
TAK/TAB	*Helicobacter pylori*	CagA	Enhances the activity of TAK1	Lamb *et al.*, 2009
*E. coli*	NleE	Methylates TAB2 and TAB3, thereby inhibiting the UBD binding ability	Zhang *et al.*, 2011b
IKK complex	*Y. pseudotuberculosis*	YopJ	Acetylates the activation loop of IKKα and IKKβ, thereby preventing their phosphorylation-induced activation	Mittal *et al.*, 2006
*Shig. flexneri*	IpaH9.8	E3 ligase that polyubiquitinates NEMO, resulting in its degradation	Ashida *et al.*, 2010
*E. coli*	NleE	Inhibits IKK phosphorylation and activation	Nadler *et al.*, 2010
IκB	*L. pneumophila*	LegK1	Ser/Thr-kinase-like protein that phosphorylates IκB	Ge *et al.*, 2009
*Shig. flexneri*	OspG	Inhibits ubiquitin-conjugating enzymes, thereby preventing ubiquitination of IκB	Kim *et al.*, 2005
*Sal. typhimurium*	AvrA	Deubiquitinates IκB	Ye *et al.*, 2007
*Y. pseudotuberculosis*	YopJ	Deubiquitinates K48-IκB	Zhou *et al.*, 2005
*Chlamydia trachomatis*	*Chla*Dub1	Deubiquitinates IκB	Le Negrate *et al.*, 2008
NFκB	*Shig. flexneri*	OspF	Dual phosphatase that prevents histone H3 phosphorylation in a subset of κB responsive genes	Arbibe *et al.*, 2007
*E. coli*	NleE	Prevents nuclear translocation of p65 and c-Rel	Newton *et al.*, 2010
*Bo. pertussis*	BopN	Promotes translocation of repressive p50	Nagamatsu *et al.*, 2009
*Ba. thetaiotaomicron*		PPAR-γ mediated export of RelA to cytoplasm	Kelly *et al.*, 2004
*E. coli*	NleC	Degrades p65	Yen *et al.*, 2010; Pearson *et al.*, 2011; Baruch *et al.*, 2011; Mühlen *et* al., 2011
*E. coli*	NleH1	Inhibits IKKβ-mediated phosphorylation of RPS3 Ser209, thereby preventing its nuclear translocation	Wan *et al.*, 2011
*E. coli*	NleC	Degrades the p300 co-activator	Shames *et al.*, 2011

Recruitment of the TAB/TAK complex to the upstream protein TRAF6 (Fig. 2) depends on the ability of TAB to recognize and bind to the K63-polyubiquitination that appears after stimulation by PAMPs. Bacteria have also evolved a mechanism to interfere with the binding properties of TABs, thereby preventing the recruitment and activation of the TAB/TAK complex. For example, EPEC-encoded NleE was found to harbour a *S*-adenosyl-l-methionone-dependent methyltranferase activity. NleE specifically methylates the zinc-coordinating cysteine in the Npl4 zinc finger domains of TAB2 and TAB3. The methylation of the cysteine residue of the TABs inhibits their ability to bind to the ubiquitin chain, and the signalling pathway is dampened (Zhang *et al.*, 2011b).

#### Can bacterial effectors interfere with IKK complexes?

Downstream of the IRAKs and TRAF6 are the TAK/TAB complexes that, when appropriate, activate IKK complexes (Fig. 2). *Yersinia* YopJ also has acetyltransferase activity, and the protein can acetylate IKKα and IKKβ on a threonine residue within their activation loop, thereby preventing their modification and activation by phosphorylation. This results in an inactive IKK complex which then dampens the signal to NFκB even in presence of PAMPs (Mittal *et al.*, 2006).

The IKK complex is also targeted by other bacteria. *Shigella* encode a bacterial E3 ubiquitin ligase IpaH9.8 that uses the A20-binding inhibitor of NFkB (ABIN-1) as an adaptor in order to K27-polyubiquitinate NEMO/IKKγ. The bacterial E3-induced polyubiquitination of NEMO/ IKKγ results in degradation of the complex, and activation of NFκB is then disturbed. Mice infected intranasally with wild-type but not IpaH9.8-deficient *Shigella* show earlier reduction of inflammation in the lung. Consistent with this, MIP-2, IL-6, IL-1β and tissue myeloperoxidase level were higher in mice infected with IpaH9.8-deficient strains. IpaH9.8 was especially functional in inhibiting NOD1-induced NFκB activation, but could also inhibit the TLR4 pathway (Ashida *et al.*, 2010). In either case, the early induction of pro-inflammatory cytokines could be inhibited in the presence of the bacterial E3 ligase IpaH9.8.

#### Do bacteria interfere with the ability of IκBα to retain NFκB in the cytoplasm?

The activation of TLR signalling results in phosphorylation followed by K48-polyubiquitination of IκBα, which targets IκBα for proteasomal destruction. The released NFκB complex can then enter the nucleus (Jiang & Chen, 2012) (Fig. 2).

One mechanism to blunt signalling at this stage is to negatively interfere with the ubiquitination process directly, that is by interfering with E1, E2 or E3 enzymes. The *Shig. flexneri* effector OspG targets the E2 ubiquitin-conjugating enzyme UbcH5, which is used by the E3 ligase complex SCF^βTrCP^. The result of the OspG–UbcH5 interaction is inhibition of polyubiquitination and subsequent degradation of IκB, resulting in suppression of the signalling pathway. The ligated ileal loop model of infection in rabbits was used to compare infection with wild-type versus an *ospG* mutant. The mutant induced stronger destruction of the mucosa than the wild-type *Shigella*, i.e. more necrotic tissue was found and the villi were almost completely eliminated. Hence the lack of *ospG* resulted in a stronger inflammatory response upon infection *in vivo* (Kim *et al.*, 2005). This clearly illustrates the effectiveness of OspG in evading induction of pro-inflammatory genes.

Another mechanism to interfere with IκB’s ability to retain NFκB in the cytoplasm is to remove the K48-ubiquitin labelling from IκB that otherwise targets it for proteasomal degradation. This mechanism is used by *Yersinia* using the deubiquitinating protease YopJ (Zhou *et al.*, 2005). The net result is that IκB remains intact and retains the ability to sequester NFκB in the cytoplasm, thereby preventing activation of NFκB responsive genes. *Sal. typhimurium* secreted factor L (SseL) has a similar function, and comparison of infection outcomes in mice showed that the presence of SseL reduced the *in vivo* inflammatory response. For example, four days post-infection the livers of mice infected with Δ*sseL* had fourfold more granulomas than livers from mice infected with wild-type bacteria. Similarly, the lumen, submucosa and surface epithelium had greater PMN (polymorphonuclear neutrophil) infiltration in mice infected with *Sal. typhimurium* Δ*sseL* compared to wild-type strain. The mice infected with the mutant strain died earlier than those infected with the wild-type strain (Le Negrate *et al.*, 2008).

Various *Salmonella* strains differ in their ability to interfere with IL-8 secretion. *Sal. typhimurium* induced IL-8 secretion in epithelial cells, while the non-pathogenic laboratory-derived *Sal. typhimurium* PhoP^c^ and naturally occurring *Salmonella pullorum* did not. The non-pathogenic strains induced phosphorylation of IκB but no ubiquitination of IκB was achieved and the pathway was blunted (Neish *et al.*, 2000). The bacterial effector is AvrA, which acts as a deubiquitinase and reduces inflammatory responses as well as host cell apoptosis (Ye *et al.*, 2007; Collier-Hyams *et al.*, 2002).

In contrast to the above examples where the bacterial effector negatively interferes with the signal cascade, the *Legionella pneumophila* effectors LnaB and LegK1 do the opposite (Ge *et al.*, 2009; Losick *et al.*, 2010). *L. pneumophila* is a facultative intracellular pathogen that infects human alveolar macrophages and epithelial cells in the respiratory tract, leading to a severe pneumonia known as Legionnaires’ disease. Activation of the pathway has been suggested to positively contribute to intracellular growth by inducing genes encoding antagonists of apoptosis (Losick *et al.*, 2010). LegK1 mimics eukaryotic serine/threonine kinases and can directly phosphorylate IκB on serine-52 and serine-36, resulting in activation of an NFκB reporter (Ge *et al.*, 2009).

### Bacterial effectors may interfere with the function of NFκB

In the nucleus, the transcription factor NFκB binds to appropriate κB sites located in available promoters of various genes encoding proteins involved in immune defence (Newton & Dixit, 2012) (Fig. 2). The availability of κB sites for transcription factors can depend on various parameters such as condensation of the DNA. Eukaryotic DNA is wrapped around four core histones (H2A, H2B, H3 and H4). The histones have an N-terminal part that protrudes out of the nucleosome, and is available for post-translational modifications. These modifications determine whether the DNA is available for transcription, i.e. phosphorylation of histone 3 (H3) increases the availability of κB sites in the promoter of IL-8. OspF from *Shig. flexneri* is a dual specific phosphatase that localizes to the nucleus where it dephosphorylates and thereby inactivates the MAPK kinases involved in H3 phosphorylation. This results in reduced H3 phosphorylation in the *IL-8* gene resulting in reduced binding of NFκB and the RNA polymerase II complex to the DNA. OspF also restricts bacterial invasion and prevents PMN recruitment in a ligated ileal loop rabbit model of infection (Arbibe *et al.*, 2007).

RelA/p65, RelB and c-Rel have a transactivation domain (TAD) involved in transactivation of target genes. In contrast, p50 and p52 can bind κB sites but lack the TAD, resulting in transcriptional repression (Li & Verma, 2002). Therefore, another strategy to modulate transcription is to interfere with which type of subunit of NFκB actually enters the nucleus. EPEC and enterohaemorrhagic *E. coli* (EHEC) encoding NleE were found to inhibit nuclear translocation of transactivating subunits p65 and c-Rel, but not the repressor p50, leading to reduced IL-8 production (Newton *et al.*, 2010). This suggests that one putative mechanism to disturb the activity of NFκB is to interfere with which subunits enter the nucleus (Fig. 2). A similar strategy has been used by *Bordetella pertussis*. These bacteria encode BopN that promotes the translocation of the repressive p50 subunit. The translocation of the repressive subunit resulted in increased expression of the anti-inflammatory IL-10, which may be an advantage for persistent colonization (Nagamatsu *et al.*, 2009). Another mechanism is to degrade subunits containing TADs. EPEC- and EHEC-encoded NleC is a zinc-dependent protease that degrades NFκB protein p65, resulting in reduced IL-8 secretion (Yen *et al.*, 2010; Pearson *et al.*, 2011; Baruch *et al.*, 2011; Mühlen *et al.*, 2011).

Appropriate localization of NFκB is needed for its function. IκB contains a nuclear export signal (NES), and shuttles between the nucleus and cytoplasm. Binding of IκB to nuclear NFκB hides the NLS signal of the transcription factor and the NES signal is exposed. This leads to nuclear export receptor exportin-1-mediated transport of the IκB–NFκB complex from the nucleus to the cytoplasm (Huang *et al.*, 2000). One anti-inflammatory strategy used by bacteria is to target the localization of NFκB subunits. *Bacteroides thetaiotaomicron* is a prevalent anaerobe of the human intestine which can attenuate inflammation caused by the pathogenic *Salmonella enterica* serovar Enteritidis. Both bacteria induce expression of peroxisome proliferator activated receptor-γ (PPAR-γ) in the host cells. Presence of *Ba. thetaiotaomicron*, in addition to *Salmonella enteritidis*, triggered a physical interaction between RelA and PPAR-γ, and nuclear export of both proteins. The interaction was dependent on the C-terminal ligand-binding domain of PPAR-γ while the transport was independent of exportin-1. However, the precise mechanism of PPAR-γ/RelA export is unknown (Kelly *et al.*, 2004).

Recently, ribosomal protein S3 (RPS3) was found to be an essential part of certain native NFκB complexes. RPS3 interacts with p65 and increases the DNA binding ability of the complex (Wan *et al.*, 2007). RPS3 directs the transcriptional complex to certain cognate κB sites, resulting in induced expression of immunoglobulin κ light chain in B cells, and cell proliferation and cytokine secretion in T cells (Wan & Lenardo, 2010; Wan *et al.*, 2007). Activation of the NFκB pathway can induce IKKβ-mediated phosphorylation of RPS3 at serine 209. This phosphorylation enhances the association of RPS3 with importin-α, which is required for nuclear translocation of RPS3 (Wan *et al.*, 2011). The virulence protein NleH1 from EHEC and EPEC interacts with and inhibits the phosphorylation of RPS3 both *in vitro* and *in vivo* thereby blocking RPS3 nuclear translocation and function (Fig. 2). (Gao *et al.*, 2009; Wan *et al.*, 2011) Gnotobiotic piglets infected with Δ*nleH1* strain had a robust inflammatory response, but reduced bacterial colonization and little diarrhoea, suggesting that NleHI might be an advantage for the bacterium in colonization and transmission (Pham *et al.*, 2012).

NFκB can influence transcription by recruitment of co-activators such as the host acetyltransferase p300/CBP. These co-activators can enhance the transcriptional activity of NFκB by various mechanisms. The co-activators can acetylate histone tails causing them to lose their electrostatic interaction with DNA, which is an advantage for transcription. Additionally, p300 can regulate the NFκB protein RelA/p65 by acetylation (Chen *et al.*, 2002). Therefore, it may not be so surprising that microbes also try to attack co-activators as a strategy for immune evasion. The zinc-dependent protease NleC from *E. coli* has recently also been found to interact with p300, causing its degradation. Overexpression or depletion of p300 in mammalian cells resulted in increased or reduced IL-8 expression, respectively (Shames *et al.*, 2011). The NleC-induced degradation of p300 may therefore contribute to an altered cytokine response.

### Conclusions and future perspectives

Microbes are recognized and eradicated by the host immune system. In order to either survive as commensals or to establish successful infections, several microbes have evolved a wide range of immune evasion strategies. Here, we have provided examples showing that bacteria can evade almost every single step in the canonical NFκB pathway. Some bacteria encode several independent effectors that can block the PRR–NFκB pathway on multiple levels to ensure silencing of immune responses (Table 1). Others produce individual effectors that target several steps in the pathway, thereby overriding the PAMP-induced signalling (Table 1). The mechanism of action varies. Many effectors mimic the function of well-known host proteins, while others act as decoys for receptors or specific inhibitors of the canonical pathway, or the bacteria may by its presence be able to induce expression of endogenous inhibitors of the pathway within the host cell (Biswas *et al.*, 2011). In any case, the net result is hijacking of the host TLR–NFκB signalling.

Hijacking the TLR–NFκB pathway can be beneficial for commensals when establishing their niche in the host (Neish *et al.*, 2000). Bacterial interference with host signalling can also be beneficial for infection. During acute infection, the hijacking results in increased bacterial reproduction within the host before recognition by immune cells. This strategy is seen among pathogenic strains of, among others, *E. coli*, *Shigella*, *Klebsiella pneumoniae* and *Salmonella* (Cirl *et al.*, 2008; Ashida *et al.*, 2010; March *et al.*, 2011; Newman *et al.*, 2006). Many of the aforementioned pathogens infect the intestine, and the integrity of the intestinal layer is of importance for the health and survival of the host. The presence of bacterial effectors that negatively interfere with inflammation may protect the bacteria from the immune response, but may also protect the host intestine from inflammatory destruction of the tissue. By this mechanism, the bacteria increase their chance of spread through diarrhoea (Kim *et al.*, 2005; Le Negrate et al., 2008; Pham *et al.*, 2012). Hijacking the TLR–NFκB pathway may also be beneficial during chronic infection. In periodontitis, the expression of CXCR4 is elevated, and targeted by the Pg-fimbriae of *Po. gingivalis* resulting in inhibition of TLR2-induced NFκB activation. Moreover, *Po. gingivalis* induces expression of the endogenous negative regulator IRAK-M. The two mechanisms for escaping the pathway may contribute to chronic infection, when the bacteria remain persistently (Hajishengallis *et al.*, 2008). Other bacteria specifically target immune cells, and here their influence on signalling pathways may be of particular importance. Stimulated dendritic cells will mature and express surface molecules that stimulate T-cells in the lymph node. *Brucella abortus* can invade and replicate within dendritic cells, where the *Br. abortus*-encoded Btp1 inhibits NFκB activation and disturbs DC maturation, resulting in reduced ability to stimulate T-cells (Salcedo *et al.*, 2008). The net outcome is beneficial to the bacteria.

Most of the intracellular effectors shown in Table 1 are produced by Gram-negative bacteria which inject the effector molecule(s) into the host cell through their type III or IV secretion system (Newton *et al.*, 2010; Lamb *et al.*, 2009; Ge *et al.*, 2009). However, patients with MyD88- or IRAK4-deficiencies are highly susceptible to infections by Gram-positive bacteria, while they have normal resistance to Gram-negative bacteria and other pathogens (von Bernuth *et al.*, 2012). Moreover, mice deficient of or with modified versions of TLR2, TIRAP, MyD88 and/or IRAK4 were more susceptible to infection with *Staph. aureus*, *En. faecium* and/or *Strep. pneumoniae* infection (Takeuchi *et al.*, 2000; Hoebe *et al.*, 2005; Deshmukh *et al.*, 2009; Leendertse *et al.*, 2008; Pennini *et al.*, 2013; Albiger *et al.*, 2005). This suggests that the MyD88/IRAK-4 pathway is of particular importance for defence against Gram-positive organisms, and it might not be a surprise if Gram-positive organisms also target this pathway as an immune evasion strategy. However, this remains to be elucidated.

Increased understanding of bacterial immune evasion mechanisms targeting TLR–NFκB signalling may be of great medical interest. Activation of the canonical NFκB pathway is important in defence against invading micro-organisms. However, aberrant activation of the signalling can be part of inflammatory and autoimmune diseases or cancer (Jeong & Lee, 2011; Han & Ulevitch, 2005; Zhu & Mohan, 2010). Microbes have evolved strategies to avoid activation of the pathway and the bacterial effectors may themselves, or in modified versions, have therapeutic potential against the above-mentioned diseases. The first example involves *Propionibacterium acnes*, which is thought to be involved in creating inflammation in acne vulgaris, a skin disease especially common among teenagers. A recent *in vitro* study showed that the *En. faecalis* bacteriocin CBT-SL5 negatively interfered with *Pr. acnes*-induced inflammation of keratinocytes (Lee *et al.*, 2008). The molecular mechanism was not addressed, but the net effect was that a bacterial molecule inhibited inflammation. Continuing and future testing may result in a useful anti-inflammatory agent. Another example involves peptides that mimic the BB-loop in the TIR domain of MyD88. Such peptides were found to attenuate staphylococcal enterotoxin B-induced pro-inflammatory cytokine production and toxicity in mice (Kissner *et al.*, 2011). Instead of using purified bacterial protein as an anti-inflammatory agent, another strategy may be to create bacteria that have an anti-inflammatory effect on certain host tissues. This strategy was tested in a mouse model of inflammatory bowel disease. Here, oral administration of lactic acid bacteria expressing the anti-inflammatory elafin had an anti-inflammatory effect in the intestine (Motta *et al.*, 2012). In summary, increased understanding of bacterial immune evasion strategies may provide new options for creating drugs to use in treatment of inflammatory diseases in the future.
